# Code blue protocol: observation and analysis of results over last 3 years in a new tertiary care hospital

**DOI:** 10.1186/2197-425X-3-S1-A205

**Published:** 2015-10-01

**Authors:** SK Pattnaik, B Ray, J Nayak, A Prusty, S Sinha

**Affiliations:** Apollo Hospitals, Bhubaneswar, India

## Intr

Hospital Emergency Codes are used in hospitals worldwide to alert staff to various emergencies. The use of codes is intended to convey essential information quickly and with minimal misunderstanding to staff, while preventing stress and panic among visitors to the hospital. A Code Blue is the term used to alert the Code Blue team (Resuscitation team) to an area where a person has had a cardiac/respiratory arrest. We in our hospital have intensive care unit personnel (Resident doctor, Nurse and Technician) assigned to attend the code blue calls.

## Objectives

The purpose of this study is to evaluate the policy, standardize and analyze results of code blue over last 3 years of its implication in our hospital setting.

## Methods

A retrospective chart analysis of all code blue cases done, noting down the cause for code blue, time to reach the spot, resuscitation measures& outcome over last 3 years.

## Results

We had 192 code blue calls in the study period over 3 years, with a monthly average of 4-5 calls. Nephrology patients had maximum number of calls (33.33%) followed by cardiology patients (14.35%). Most of the code blue calls (72.46%) were during off times (8PM-8AM). Bradycardia is the commonest cause (44%) of alert. Our code blue response time was 3 minutes. Return of spontaneous circulation achieved in 54.25% cases, of which 22.80% patients discharged home in conscious state.

## Conclusions

Code blue team has made a definite positive impact after its implementation in our hospital with improvement in patient outcome with passing time.

